# An Embedded Stress Sensor for Concrete SHM Based on Amorphous Ferromagnetic Microwires

**DOI:** 10.3390/s141119963

**Published:** 2014-10-24

**Authors:** Jesús Olivera, Margarita González, José Vicente Fuente, Rastislav Varga, Arkady Zhukov, José Javier Anaya

**Affiliations:** 1 Instituto de Tecnologías Físicas y de la Información “Leonardo Torres Quevedo”, ITEFI (CSIC), Madrid 28006, Spain; E-Mails: m.g.hernandez@csic.es (M.G.); jj.anaya@csic.es (J.J.A.); 2 Instituto Tecnológico de la Construcción (AIDICO), Valencia 46980, Spain; E-Mail: jvfuente@aidico.es; 3 Institute of Physics, Faculty of Science, University of Pavol Jozef Safarik, Kosice 040 01, Slovakia; E-Mail: rastislav.varga@upjs.sk; 4 Dpto. Física de Materiales, Facultad de Química, UPV/EHU, San Sebastián 20018, Spain; E-Mail: arkadi.joukov@ehu.es; 5 IKERBASQUE, Basque Foundation for Science, Bilbao 48011, Spain

**Keywords:** concrete, embedded sensor, ferromagnetic microwires, switching field, SHM

## Abstract

A new smart concrete aggregate design as a candidate for applications in structural health monitoring (SHM) of critical elements in civil infrastructure is proposed. The cement-based stress/strain sensor was developed by utilizing the stress/strain sensing properties of a magnetic microwire embedded in cement-based composite (MMCC). This is a contact-less type sensor that measures variations of magnetic properties resulting from stress variations. Sensors made of these materials can be designed to satisfy the specific demand for an economic way to monitor concrete infrastructure health. For this purpose, we embedded a thin magnetic microwire in the core of a cement-based cylinder, which was inserted into the concrete specimen under study as an extra aggregate. The experimental results show that the embedded MMCC sensor is capable of measuring internal compressive stress around the range of 1–30 MPa. Two stress sensing properties of the embedded sensor under uniaxial compression were studied: the peak amplitude and peak position of magnetic switching field. The sensitivity values for the amplitude and position within the measured range were 5 mV/MPa and 2.5 μs/MPa, respectively.

## 1. Introduction

The extensive application of concrete structures in recent years has made the observation, monitoring, and evaluation of their structural condition in real time to minimise in-depth inspections or any kind of major accidents a very important matter. For such purpose, monitoring with the use of low-cost distributed sensors could be an alternative. An effective monitoring action can not only keep under control an increasing number of concrete infrastructures with only a minimal increase in effort, but will also reduce (and for some aspects, remove) the cost of inspection while offering a higher level of reliability. With respect to inspections, especially in-depth ones, monitoring would not require traffic interruptions. The main goal of applying low-cost distributed sensors is the reduction of maintenance costs by using currently available wireless technologies [1].

Traditional state-of-the-art non-destructive evaluation (NDE) techniques with attached sensors have some insuperable disadvantages: the necessity of external connections, small coverage area, complicated signal processing, and quite strong local mechanical distortions introduced by the sensor elements. Much progress in concrete science is expected in the near future with the adaptation of new knowledge generated by the quickly growing field of micro/nanotechnology. However, because nano-sensors have extremely small dimensions, their exploration and fabrication require access to sophisticated and sometimes expensive technology [2]. To simplify the fabrication procedure and reduce sensor costs, alternative fabrication techniques need to be explored [3].

Compressive strength is the most common performance measure for concrete. Tests are usually carried out in the laboratory by loading cubic or cylindrical samples in a compression testing machine until failure [4]. Ultrasonic pulse velocity or rebound hammer measurements will give qualitative results (calibration is required) and are used in the laboratory or on site.

Various techniques have been applied to detect the internal stress/strain in concrete structures. Strain gauges, which are basically adhered to the outer surface of a specimen, are capable of measuring pressure, force and tension, converting the units into measurable electrical resistance at low cost. The value and distribution of strain inside a concrete specimen under axial loading are not equal to those obtained at a point on the surface of the specimen. Information on different cement-based sensors embedded as simple aggregates to obtain data on the internal mechanical stress in different parts of the structure is necessary for a better understanding of the behaviour of this heterogeneous material. Large concrete structures tested by surface strain gauges require more complex analysis that includes observing time-history records for damage location and long-time durability predictions [5]. Currently, these gauges are used to monitor strains in mass concrete blocks and reinforced concrete slabs in the construction sector, especially in health monitoring. The electrical (resistance) strain gauges (ESG), linear variable differential transformers (LVDT), and the vibrating wire strain gauges (VWG) are commonly used [6,7]. Also fibre optic sensors [8] and piezoelectric sensors [9] have been shown to have a good performance in measuring internal stresses and deformation.

Most commonly, the positioning of the mechanical stress/strain sensors in reinforced concrete elements is determined by attaching sensors to the reinforcement. However, the degree of reinforcement in massive concrete elements is relatively low, and reinforcement is mostly concentrated in near-surface areas.

All the sensing techniques that rely on magnetic, piezoelectric, and optical approaches have their own specific merits and disadvantages. However, large-scale applications in civil structures require the development of innovative distributed sensors that are able to fulfil most of the following requirements: accuracy, robustness, price, immunity to magnetic interference, easy positioning, simplicity of operation, surface bonding between the sensor and the cement structure, and remote operation. To satisfy this need, magnetic sensors based on cement-based composites with embedded magnetic microwires were developed.

### Magnetic Microwires

**Figure 1.**
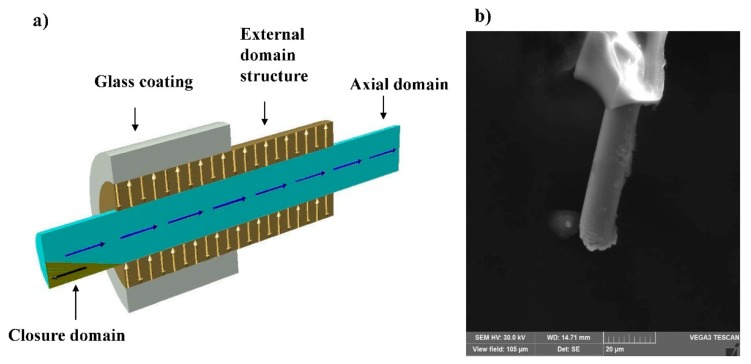
(**a**) Schematic domain structure of positive magnetostriction microwire; (**b**) Micrograph of the glass-coated microwire.

Amorphous magnetic microwires have attracted much attention due to their tiny dimensions (around 1–100 μm in diameter), enabling the possibility of tailoring their magnetic and mechanical properties by a specific method of fabrication, appropriate treatments, and simplified measurement [3]. These microwires (see photo in Figure 1b) consist of a metallic nucleus (10–80 μm in diameter) covered by a glass coating (2–20 μm thickness). They are produced by simultaneously melting the metallic nucleus with the insulating coating and then rapidly cooling it in water to achieve an amorphous structure with a unique distribution of strong internal mechanical stresses. Due to the lack of magnetocrystalline anisotropy in the amorphous state, magnetoelastic and magnetostatic anisotropies determine the domain structure and magnetic properties of the microwires. The mechanical stresses created during their fabrication, together with magnetostriction, determine the strength of magnetoelastic anisotropy. The details of their properties can be found in [2].

The domain structure of amorphous microwires with positive magnetostriction consists of one single axial domain in the inner part of the microwire, which is surrounded by a radial domain structure just below the surface [10], as illustrated in Figure 1a. Additionally, small closure domains appear at the ends of the microwire to decrease the stray fields [11].

**Figure 2.**
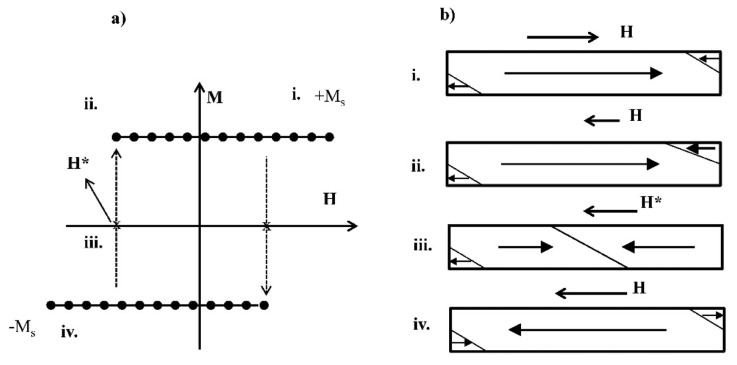
(**a**) A schematic representation of the domain structure at the inner core during different stages of the magnetization process; (**b**) The corresponding states in the hysteresis loops.

Glass-coated ferromagnetic microwires exhibit unusual and interesting magnetic properties such as magnetic bistability and giant magneto-impedance effect [3]. The magnetic bistability of microwires with positive magnetostriction is characterized by the appearance of rectangular hysteresis loops at a low applied magnetic field (see Figure 2a). The origin of the squared hysteresis loops of the microwire is interpreted in terms of nucleation or depinning of the reversed domains inside the internal single domain and the consequent domain wall propagation [12].

Figure 2b shows schematically the domain structure during the magnetization reversal process inside the inner core of a bistable microwire. The arrows indicate the magnetization orientation within each magnetic domain.

The change of magnetization takes place when an axial magnetic field H is gradually increased in the opposite direction from the stable state (i), the magnetization remains close to the stable state, and then the magnetization reverses its orientation within the core at the critical or switching field H*, as only one Barkhausen jump from the magnetic wall formed at the end of the microwire, to finally reach another stable state in (iv). The static magnetization of such microwires has just two values (+M_s_ and −M_s_) [13], and the switching between these two states occurs when the external field reaches the value of the so-called switching field H* (see Figure 2a).

Because the magnetic properties of amorphous glass-coated microwires are determined by the magnetoelastic interaction of the magnetic moment with the mechanical stresses, the switching field is very sensitive to the applied mechanical stress [14,15]. Due to the different thermal expansion coefficients of the metallic nucleus and glass coating, additional stresses appear inside the metallic nucleus; hence, the switching field is sensitive to temperature, too.

Apart from the magnetoelastic contribution to the switching field, there exists another contribution that arises from the structural relaxation of amorphous microwires. This contribution is sensitive not only to temperature, but also to the frequency of the exciting magnetic field. Hence, by setting the excitation frequency, one can choose to measure either the temperature or the applied mechanical stress [16]. The stress dependence of the switching field in carbon fibre composites with embedded microwires has already been studied [17], and the use of embedded microwires in titanium-based implants has been proposed [18].

On other hand, the results of a study of giant magneto-impedance effect on the glass fiber-reinforced composites containing continuous magnetic microwires have revealed that they are very promising for applications in structural health monitoring and stress sensing [19].

The dynamics of domain wall propagation can be very effectively modified by the different anisotropies introduced into the microwire by a magnetic field [20], mechanical stress [21], thermal treatment [22], *etc*.

Due to their complex anisotropy distribution, microwires have very fast domain wall propagation with the domain wall velocities of up to 18,000 km/s [12], which gives us an opportunity to obtain induced signals even from a small volume of single microwires. The domain wall dynamics in glass-coated microwires is strongly dependent on external parameters, such as magnetic field, temperature, mechanical stress, and so on [16]. Such variations increase the application potential of the microwire.

This leads us to the idea of using microwires in the construction of a miniaturized multifunctional sensor for structural health monitoring purposes. Consisting of a single microwire embedded in cement-based composite, this sensor can measure the state of the internal stress of concrete by means of electromagnetic induction. Its versatility comes from its simple and cheap method of preparation, efficiency (up to a few kilometres of microwire can be produced from 1 gram of master alloy [3]), insulation against the alkali environment of concrete through an adequate composition of its glass-coating, small dimensions to avoid any disturbance to its stress state and the possibility of remote monitoring.

In the current paper, a new design of smart concrete aggregate as a candidate for applications in structural health monitoring of critical elements in civil infrastructure is presented. The smart concrete aggregate is a cement-based stress/strain sensor which has been developed by utilizing the stress/strain sensing property of an embedded magnetic microwire in cement-based composite (MMCC).

## 2. Experimental Section

### 2.1. MMCC Sensor

The MMCC sensors were made of magnetic microwires embedded in cement-based composite. The use of magnetic microwires allows creating a built-in stress/strain sensor inside the material without affecting its mechanical behaviour.

#### 2.1.1. Fabrication

The study has been performed on glass-coated amorphous microwires with a nominal composition of Fe_75_B_9_Si_12_C_4_ (magnestostriction constant of λ_s_ ≈ +30 × 10^−6^), fabricated according to the Taylor-Ulitovsky method [3]. The fabrication of glass-coated microwires involves the rapid solidification of a composite microwire consisting of a ferromagnetic metallic nucleus inside a glass coating. Large internal stresses are induced inside the metal core during the production process, and these stresses significantly determine the magnetic behaviour of the microwire [23].

The microwire length was 60 mm. In an alkaline concentrated environment, such as concrete (pH > 13 in most cases), the glass-coating of the microwire can provide moderate protection against metallic core deterioration by hydroxide ions (OH^−^). The composition of the Pyrex-like glass coating can be specified with a high percentage of zirconia (ZrO_2_), which enhances its resistance to alkali in cement composites [24].

**Figure 3.**
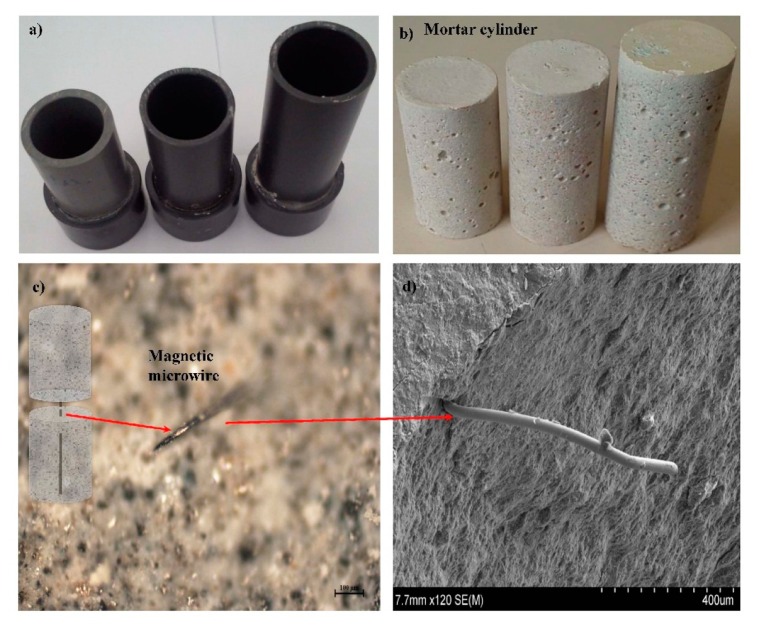
(**a**) Molds for MMCC sensors; (**b**) mortar cylinder with an embedded microwire; and (**c**) and (**d**) optical and electronic micrograph, respectively, of the embedded microwire.

The magnetic microwire was inserted into the axis of a mortar cylinder. The cylinder geometry was chosen because the calibration of the sensor is simple. The mortar cylinders can be fabricated with different length-to-diameter ratios, as shown in Figure 3a,b. It is worth mentioning that, to maintain the sensing property (bistability) of the highly magnetostrictive microwires, a minimal length of more than a few millimetres is needed [25]. Figure 3c,d shows an embedded magnetic microwire in the transverse plane in the sectioned mortar cylinder.

The mix proportions of the mortar are shown in Table 1. The mix proportions of the cement-based composite were designed to obtain a similar compressive strength as the aggregates used. The mortar was fabricated and stored in molds for 24 h; and after demolding, the mortar was cured by immersion at 20 °C for 28 days. After 28 days of fabrication, the sensors were added into concrete as a simple extra aggregate. In this work, the mortar cylinder was fabricated with a diameter of 25 mm and length of 64 mm of length.

**Figure 4.**
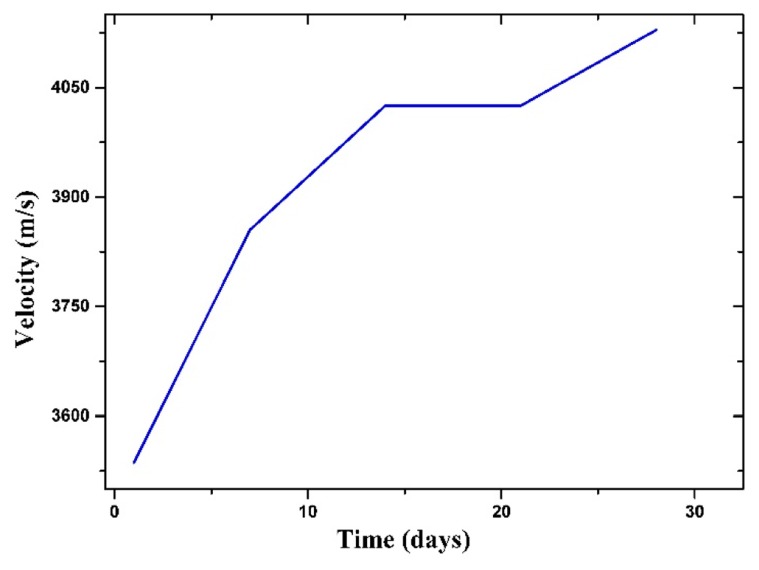
Monitoring the mortar cylinder curing process through the measurement of ultrasonic velocity over time.

Further, 40 × 40 × 160 mm^3^ prismatic mortar specimens with the same proportions as the cylinders were made to determine the compressive strength of the material at 28 days. The evolution of the mechanical properties of the mortar was monitored during the curing process by measuring the ultrasonic velocity with the use of Pundit equipment, transducers with of 500 kHz operation in transmission mode, and medical gel as couplant. The measurements were made at days 1, 3, 7, 14, and 28 of the curing process (see Figure 4); the velocity was determined by conventional methods based on the propagation time through the samples. Based on the figure, which shows the mean velocity at each time the velocity increases with curing time, indicating the development of compressive strength [26].

#### 2.1.2. Bistability and Design of MMCC Sensors

**Figure 5.**
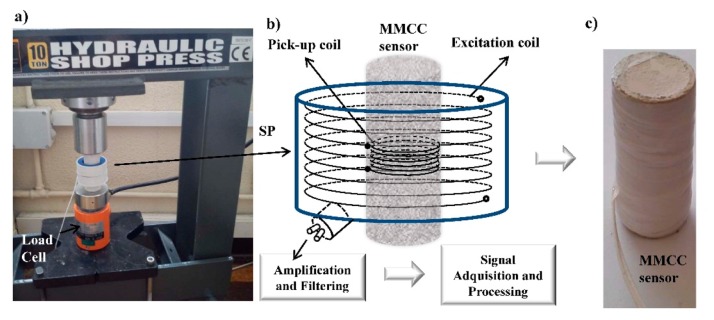
(**a**) Hydraulic press machine used to apply the mechanical compression force; (**b**) signal probe (SP) and (**c**) MMCC sensors ready to be embedded.

We have slightly adapted the Sixtus-Tonks method [27] for the calibration of MMCC sensors. We used a signal probe (SP) with concentric coil geometry as a previous step to the characterization and design of the coil systems for each MMCC sensor. The excitation and pick-up coils were located inside a casing of non-conductive material (Figure 5b), which was hollow inside to accommodate the MMCC sensor. The excitation coil was fed with sinusoidal shape current and the pick-up coil to detect the feedback of the MMCC sensor by a simple electromagnetic induction. This contact-less method can be used to determine the magnetic properties, on which this sensor is based, *i.e.*, magnetic bistability and the stress dependence of the switching field.

Load was applied with a hydraulic press, as shown in Figure 5a. The load cell was used to control the applied compressive mechanical force to the MMCC sensors below its breaking force. A signal probe (SP) (Figure 5b) was employed to obtain the response of the embedded microwire as a function of mechanical compression stress prior to beginning the MMCC sensor design. The excitation field in the MMCC sensor (Figure 5c) was made by winding a coil of copper on the mortar cylinder (Figure 5b); the pick-up coil was then wound on the former winding. Moreover, the windings were protected with Teflon. Figure 5c shows the MMCC sensors ready to be embedded.

The excitation winding is connected to the power supply to generate the excitation field that magnetizes the microwire, which causes the propagation of a magnetic domain wall along the microwire. The pickup coil captures the induced signal when the domain wall passes through it; this signal can be amplified and filtered to obtain the MMCC sensor signal. A digital oscilloscope captures the output of the pickup coil with the excitation signal as reference. After adequate processing and filtering, signal processing at a sampling rate of 2.5 MHz was carried out. In all experiments, an excitation field frequency of 600 Hz excitation field was used, and the amplitude was set to 900 A/m. All measurements reported here were taken at room temperature.

**Figure 6.**
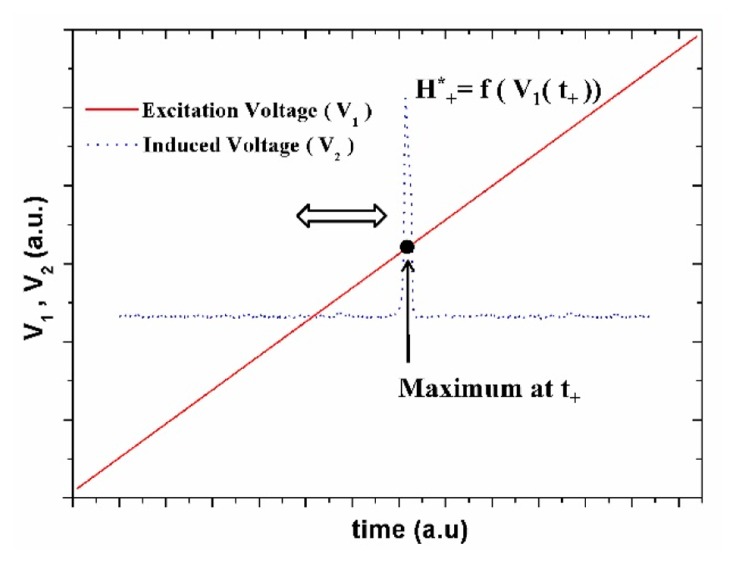
Sharp maximum (blue dot) appears at the end of the pick-up coil when the external field (red line) approaches the switching field.

As described above, the pick-up coil is able to detect the motion of a magnetic domain wall represented as an induced voltage peak (see Figure 6) when the external magnetic field caused by the excitation current exceeds the magnetic switching field H*. A voltage peak, maximum or minimum depending on the sense of the excitation magnetic field, is generated when the domain wall starts moving in the sensing pick-up coil. Measurement of the characteristics of the peak is performed through digital comparison with the time of the excitation (V_1_) and induced (V_2_) voltage signal. Therefore, the time at which the maximum (t_+_) or minimum (t_−_) induced signal takes place fixes the voltage peaks (V_+_* and V_−_*) of the excitation signal at which the domain wall propagation occurs. The corresponding switching fields H*_+_ and H*_−_ can be easily determined from the value of the current through the excitation coil that creates the magnetic field. The switching field H*_+_ can be estimated from the sharp peak voltage (maximum) position, as shown in Figure 6.

The sensor can be influenced by the local metallic materials, or the surrounding magnetic fields, such as in reinforced concrete. It is possible to remove stray magnetic fields resulting from both the Earth's magnetic field and the surrounding metallic materials. In such case, the switching field must be measured in both directions of the applied excitation magnetic field.

Thus a reverse magnetic field H*_+_ is measured when the excitation magnetic field increases, and H*_−_ when the excitation magnetic field decreases. Finally, the switching field immune to parasitic magnetic fields, H*, is proportional to the difference of two components [17]:

(1) $$H^{*}=\frac{H_{+}^{*}-H_{-}^{*}}{2}$$

whereas the local parasitic magnetic field is given by the sum of the two components [17]. As a result, the determination of the switching field may be done even with the influence of any local fields.

### 2.2. Concrete Specimen Design, Sensor Location and Testing

**Figure 7.**
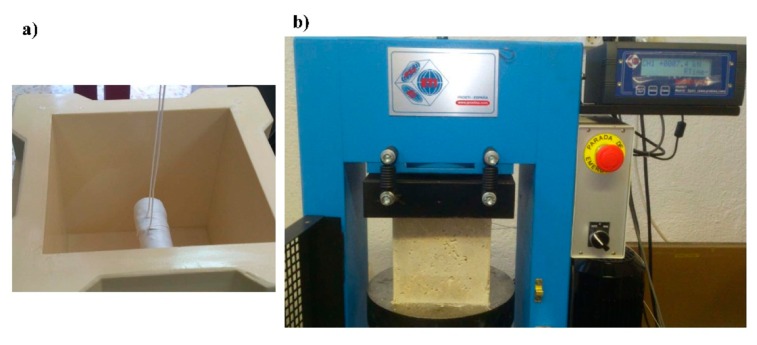
(**a**) MMCC sensor located in the centre of a normalized cubic mold; (**b**) Experimental set-up for the compressive loading test.

Several concrete cubic specimens (15 cm of side) were fabricated with Portland cement. The fine aggregate was natural sand, and the coarse aggregate presented a maximum radius of 12 mm. The mix proportions are shown in Table 1. The MMCC sensor embedded in a concrete specimen was placed in a horizontal position, parallel to the base of the cubic mold and centred in height and width as shown in Figure 7a. The sensor was fixed in that position by means of two loops of nylon wire.

In this work, the measurements were carried out with embedded windings around the mortar cylinder to allow taking measurements at any depth and with the greatest possible stability. However, the primary and secondary windings of the MMCC sensor may be replaced by windings outside the concrete specimen if the microwire is located near the surface of the structure.

Compressive strength tests were done on cubic and prismatic specimens of concrete and mortar, respectively, by using a compression machine (3000 kN) supplied by Proeti S.A. (Figure 7b). The same compression machine was used to apply cyclic loading tests to concrete with embedded MMCC sensors. The compression machine exerts a constant progressing force on the cubes at a loading rate of 0.14 MPa/s. A rest period of 3 min was applied between consecutive loading tests.

## 3. Results and Discussion

### 3.1. Mechanical Behaviour of Concrete and Mortar Specimens

The values of compressive strength obtained in mortar and concrete specimens without sensor were 44 MPa and 33 MPa, respectively. The compressive strength of concrete is used to determine the rupture load limit of concrete and to establish the upper limit of cyclic load for concrete with sensor, in this case, 75% of the compressive strength.

### 3.2. Compressive Stress Sensing Property of MMCC Sensor

The as-quenched microwires showed rather sharp voltage peaks induced in the pick-up coil, which were associated with the fast magnetization switching in magnetically bistable microwires. The shape of the voltage from the pick-up coil changes under tensile stress application [14,20].

Based on the performance of the embedded MMCC sensor (see stress sensing and mechanical properties in Table 2), two stress sensing properties at the value of the switching field under uniaxial compression have been studied: peak amplitude and peak position. The compressive stress measured by the MMCC sensor was calculated based on the change in the properties of this sharp voltage peak induced in the pick-up coil.

One can observe that the relative change of the peak position is smaller than the corresponding change of the relative amplitude, reflecting the high values of internal stress arising from the specific fabrication process of the as-prepared amorphous microwire.

### 3.3. Compressive Stress Sensing Properties of Embedded MMCC Sensor

**Figure 8.**
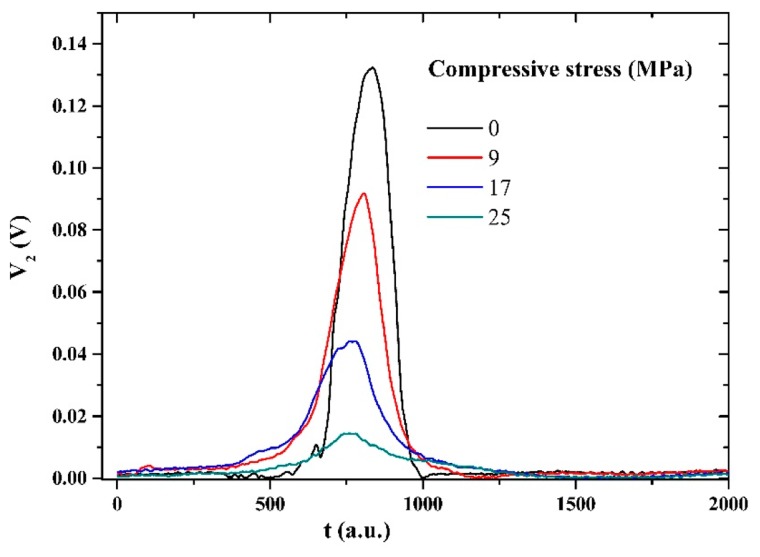
Change of shape (peak amplitude and peak position) of the voltage from the pick-up coil (V_2_) under applied compressive stress.

Let us comment on the correlation between the sensing properties of the microwire and the induced signal in the pick-up coil produced by just one domain wall passing through it. The voltage peak that appears on pick-up coil of the MMCC sensor is induced by the temporal change of magnetic flux during the remagnetization process. From the dependence of the peak features on the compressive stresses [28], we can deduce the influence of compressive stresses on the remagnetization process, as shown in Figure 8.

The compressive stresses gradually decrease the relative volume of the axially magnetized internal core of the microwire at the expense of the radial volume, which leads to a lower value of the induced voltage peak. On the other hand, the peak positions shift toward a lower stress level; therefore, the peak position decreases with the compressive stress. It is worth mentioning that the existence of high quenched-in internal stresses as a result of the fabrication process leads to a very small changes in the peak position. Additionally, as observed in Figure 8, the peak position measurement became unstable due to the large broadening of the peak at high applied mechanical compressive stress.

**Figure 9.**
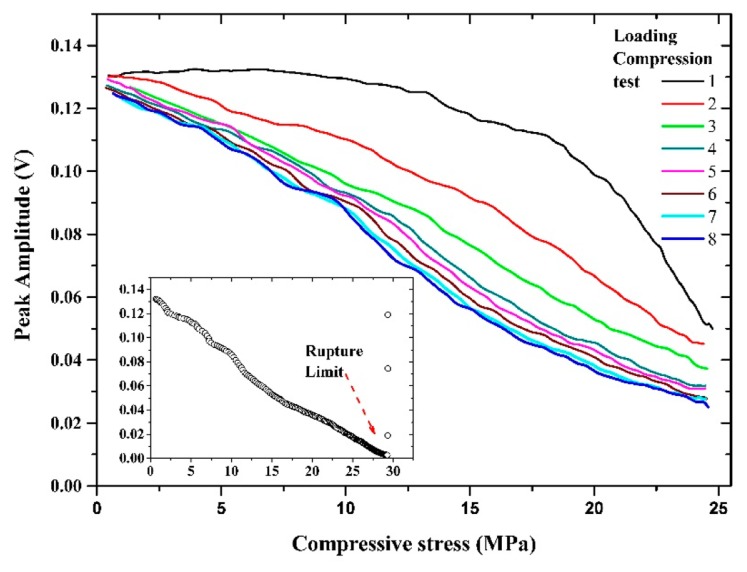
Variation of the peak amplitude of embedded MMCC sensor.

**Figure 10.**
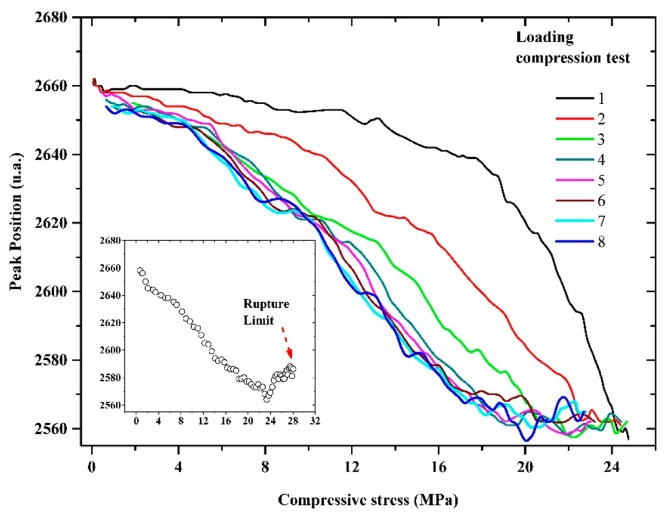
Variation of the peak position of the embedded MMCC sensor.

The variation of the stress sensing property, peak amplitude and the peak position, of the embedded MMCC sensor as a function of compressive force (9 cycles of loading), is shown in Figures 9 and 10; the inset illustrates the dependence of the sensing process up to the rupture limit.

The concrete specimen contains voids and micro-cracks in the initial state [29]. Stress redistribution in a concrete specimen exposed to cyclic loading leads to increase of the stress concentration around the weaker zones [30]. As a consequence, stabilization of the mechanical stress along the specimen takes places after a several cycles of loading. In particular, the measured stress in the concrete specimen after the 8th loading test shows a repeatable and more precise behaviour (see Figure 9).

**Figure 11.**
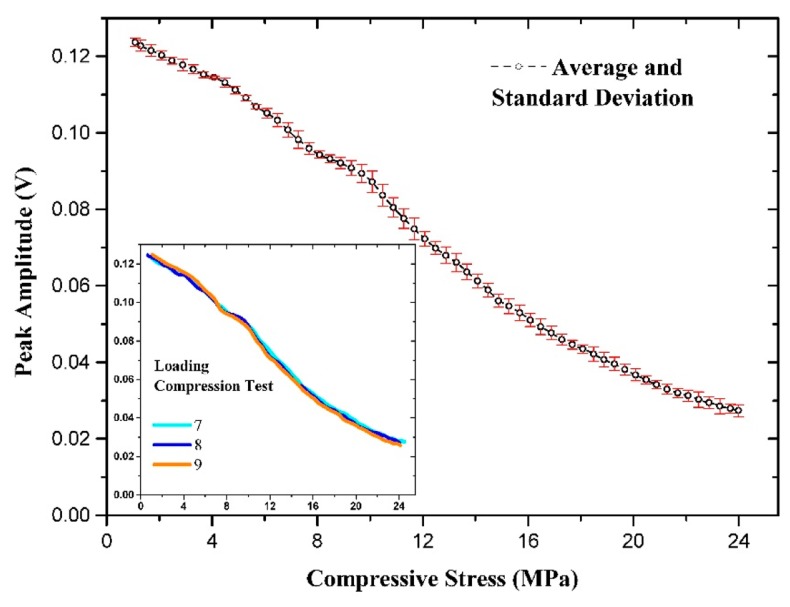
Averaged and standard deviation of the peak amplitude.

**Figure 12.**
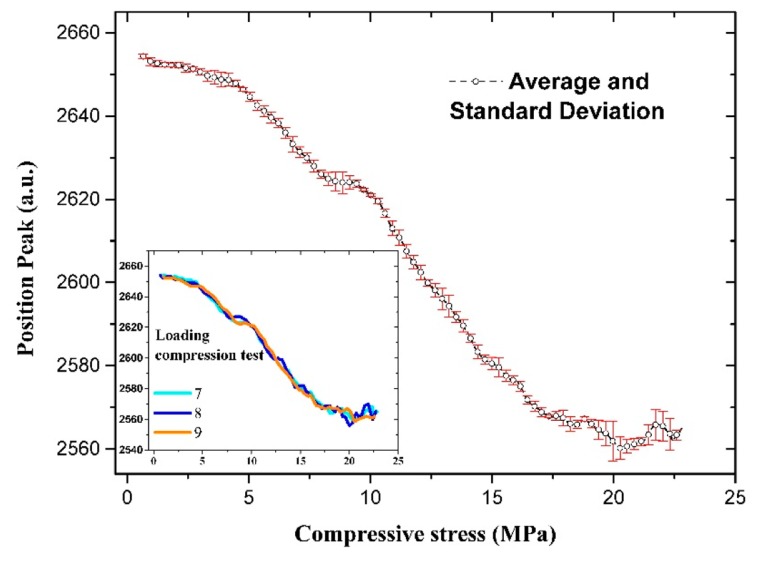
Averaged and standard deviation of the peak position.

Figure 10 shows that at high applied mechanical compressive stress, the peak position becomes unstable due to the wider half-width of the peak, as shown in Figure 8. Figures 11 and 12 show the means and standard deviations of the sensing properties in the last three cycles, during which the material was considered to be stable. As shown in the Figure, the deviation of the peak amplitude is very stable throughout the measurement range. However, the deviation of the peak position measurement increases substantially from 18 MPa because have become unstable, as shown in Figure 8.

**Figure 13.**
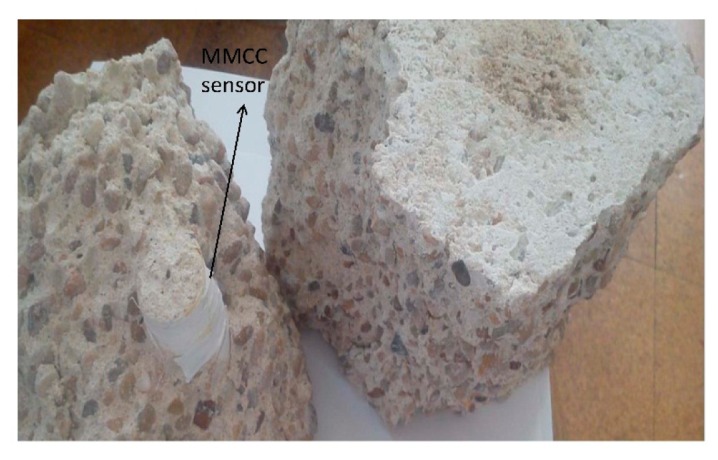
MMCC sensor obtained after the breaking of the cubic concrete specimen.

For the concrete specimen with sensor, no cracks appeared during the application of successive loading, which was applied only up to 75% of the rupture limit. Finally, when the specimen was tested until failure, macro-cracks progressively extended after 28 MPa; when the load reached about 33 MPa, the materials fails completely. However, the sensor integrity and sensing characteristics were maintained after the breaking of the specimen (Figure 13).

## 4. Conclusions

A non-destructive testing method for the determination of the mechanical resistance of concrete is presented, consisting of the stress sensitivity of the magnetization reversal of a ferromagnetic microwire. The proposed MMCC sensor was designed to study the possibility of contact-less measurement of compressive stress in a cement-based composite by using an embedded amorphous magnetic microwire. Two stress sensing properties of the switching field value of the embedded sensor under uniaxial compression were selected: peak amplitude and peak position. The sensitivity values of the amplitude and position within the application range were 5 mV/MPa and 2.5 μs/MPa, respectively. The peak amplitude allows greater precision and measurement range. The MMCC sensor under uniaxial compression shows a precise and repeatable sensing ability for compressive stress. The experimental results indicate that it is possible to obtain a signal from a microwire embedded in a concrete structure and thus to determine its internal stresses. Hence, it is possible to estimate the structural health of concrete. Their versatility and also its possible integration without physical contact make these sensors suitable and useful for several applications in field of the embedded sensors.

Knowledge about fatigue is very important both from an economic point of view and from the aspect of structural safety. Examples of concrete structures that are exposed to cyclic loading that causes fatigue are roads, bridges, wind power plants, and so on. The sensors described in this paper may provide enhanced diagnostics and damage progression prediction capabilities, as well as new capabilities for concrete structural health monitoring.

## Figures and Tables

**Table 1.** Mix proportions of mortar and concrete.

**Materials**	**Mortar**	**Concrete**
White cement I 52.5R	0.150 kg	4.85 kg
Siliceous sand	0.450 kg	11.0 kg
Gravel		11.3 kg
Water	0.0675 kg	2.55 kg
Sika Viscocrete 5990	2.2% of weight of cement	1.2% of weight of cement

**Table 2.** Stress sensing of MMCC sensor and mechanical properties.

**Stress Sensing Properties**		**Mechanical Properties**	
	
**Peak Amplitude S_A_ (mV/MPa) Range (1→30 MPa)**	**Peak Position S_P_ (μs/MPa^−1^) Range (3→20 MPa)**	**Strength (MPa)**	**Dynamic Young Modulus (GPa)**
5 ± 0.035	2.5 ± 0.4	44	36.3
